# Histone deacetylase inhibition as an alternative strategy against invasive aspergillosis

**DOI:** 10.3389/fmicb.2015.00096

**Published:** 2015-02-16

**Authors:** Frédéric Lamoth, Praveen R. Juvvadi, William J. Steinbach

**Affiliations:** ^1^Division of Pediatric Infectious Diseases, Department of Pediatrics, Duke University Medical Center, Durham, NC, USA; ^2^Infectious Diseases Service, Department of Medicine, Lausanne University Hospital, Lausanne, Switzerland; ^3^Institute of Microbiology, Lausanne University Hospital, Lausanne, Switzerland; ^4^Department of Molecular Genetics and Microbiology, Duke University Medical Center, Durham, NC, USA

**Keywords:** lysine deacetylases, *Aspergillus fumigatus*, trichostatin A, heat shock protein 90, antifungal therapy, antifungal resistance

## Abstract

Invasive aspergillosis (IA) is a life-threatening infection due to Aspergillus fumigatus and other Aspergillus spp. Drugs targeting the fungal cell membrane (triazoles, amphotericin B) or cell wall (echinocandins) are currently the sole therapeutic options against IA. Their limited efficacy and the emergence of resistance warrant the identification of new antifungal targets. Histone deacetylases (HDACs) are enzymes responsible of the deacetylation of lysine residues of core histones, thus controlling chromatin remodeling and transcriptional activation. HDACs also control the acetylation and activation status of multiple non-histone proteins, including the heat shock protein 90 (Hsp90), an essential molecular chaperone for fungal virulence and antifungal resistance. This review provides an overview of the different HDACs in *Aspergillus* spp. as well as their respective contribution to total HDAC activity, fungal growth, stress responses, and virulence. The potential of HDAC inhibitors, currently under development for cancer therapy, as novel alternative antifungal agents against IA is discussed.

## INTRODUCTION

The filamentous fungus *Aspergillus fumigatus* is the primary cause of invasive aspergillosis (IA), a frequent and life-threatening infection in immunosuppressed patients. Novel therapeutic approaches of IA are needed to overcome emerging resistance to azoles, used as first-line therapy, and the toxicity or limited efficacy of second-line treatments such as amphotericin B and echinocandins. Moreover, other *Aspergillus* spp. with less susceptibility to current antifungal drugs (*A. flavus*, *A. terreus*, *A. ustus*) account for a substantial proportion of IA.

**FIGURE 1:**
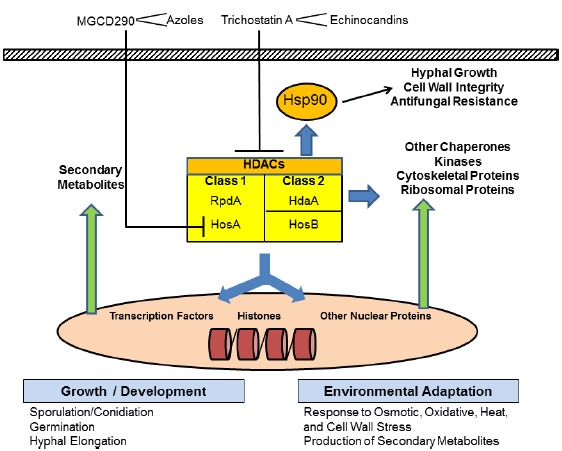
**Schematic representation of the role of the classical histone deacetylases (HDACs) in *Aspergillus fumigatus*.** Classical HDACs in *Aspergillus* spp. include RpdA and HdaA, contributing to the major part of total HDAC activity, and HosA and HosB, contributing to a minor part. RpdA and HosA belong to class 1. HdaA belongs to class 2. HosB was proposed as the only member of a distinct class. In the nucleus, HDACs deacetylate core histones, which results in chromatin remodeling and transcriptional regulation of the expression of secondary metabolites and multiple proteins. They also regulate the function of other nuclear proteins involved in DNA replication, DNA repair, nuclear transport, or cell cycle. HDACs also deacetylate and control the activation of multiple cytosolic proteins, including the heat shock protein 90 (Hsp90), which is essential for fungal growth and stress responses. HDACs thus contribute to fungal development and environmental adaptation in multiple ways. Trichostatin A and other hydroxamate analogs inhibit both class 1 and class 2 HDACs (with the exception of HosB) displaying antifungal activity against *A. fumigatus* and potentiating (<) the effect of cell wall inhibitors such as the echinocandins. MGCD290 is a specific inhibitor of HosA with poor intrinsic antifungal activity, but potentiates the effect of cell membrane inhibitors such as the azoles.

The pathogenesis of IA relies on multiple microbial and host factors. At the pathogen level, morphogenetic changes (germination, hyphal growth), thermal and metabolic adaptation to the human body, metabolite production and resistance to antifungal drugs are all determinants contributing to the virulence of *A. fumigatus* (Tekaia and Latge, 2005; Kwon-Chung and Sugui, 2013). Adaptation to environmental conditions involves processes of chromatin remodeling and transcriptional regulation. The modulation of gene expression depends on the packaging of DNA by core histones constituting the dynamic structure of chromatin. Direct DNA methylation and post-translational modifications of histones (such as acetylation and methylation) are important for conformational changes and transcriptional regulation (Brosch et al., 2008). Histone acetyltransferases (HATs) and histone deacetylases (HDACs, also referred to as lysine deacetylases, KDACs) are the enzymes responsible for the reversible process of acetylation (i.e., addition of an acetyl group to the *ε*-amino group of a lysine residue) and deacetylation of core histones, respectively. Moreover, these enzymes are also involved in the functional regulation of proteins other than core histones, including the heat shock protein 90 (Hsp90), an essential molecular chaperone for proper protein folding and maturation (Yu et al., 2002). In fungi, Hsp90 was shown to play a crucial role in morphogenetic changes, stress adaptation, virulence, and antifungal resistance, and thus represents an attractive antifungal target (Cowen, 2013; Lamoth et al., 2014a). Acetylation of Hsp90 results in impaired Hsp90 function, while HDACs reverse this process and activate the chaperone (Robbins et al., 2012; Lamoth et al., 2014c). Thus, HDACs play a role in fungal virulence by controlling the expression and function of multiple proteins, including chaperones and secondary metabolites that are important for basal growth or stress adaptation, either at the transcriptional level (by deacetylation of core histones and regulation of their expression) or at the post-translational level (by deacetylation and activation of the protein; Figure 1). This review will focus on HDACs and their link with Hsp90. The potential of HDAC inhibitors as novel antifungal therapies of IA will be discussed.

## HDACs IN *Aspergillus*

Histone deacetylases are categorized in three families: (1) the zinc-dependent or “classical” HDACs (including classes 1 and 2, as well as HOS3-like HDACs in fungi and class 4 in other eukaryotes), (2) the NAD^+^-dependent SIR2-like HDACs or sirtuins (also referred as class 3), and (3) the HD2-like enzymes (found exclusively in plants; Trojer et al., 2003; Brosch et al., 2008).

### CLASSICAL HDACs

Five classical fungal HDACs have been characterized in the model yeast *Saccharomyces cerevisiae* and divided into two classes, the class 1 or RPD3-type HDACs (including RPD3, HOS1, and HOS2) and the class 2 or HDA1-type HDACs (HDA1; Rundlett et al., 1996; Kurdistani and Grunstein, 2003). HOS3 is a fungal-specific HDAC, representing a third group distinct from the other ones, albeit initially classified within class 2 (Carmen et al., 1999). Our knowledge of classical HDACs in filamentous fungi is essentially derived from studies in *A. nidulans*, *A. oryzae*, and in plant pathogenic fungi (Brosch et al., 2008). In *A. nidulans*, two class 1 enzymes have been identified, RpdA and HosA, with high sequence similarity to yeast RPD3 and HOS2, respectively, except for a 200-amino acid C-terminal extension specific to *A. nidulans* RpdA (Graessle et al., 2000). There is no HOS1 *Aspergillus* ortholog. HdaA was characterized as the yeast HDA1 (class 2) ortholog (Trojer et al., 2003). A HOS3 ortholog, HosB, was also identified (Trojer et al., 2003). Putative orthologs for all these enzymes were identified in *A. oryzae* and *A. fumigatus* (Trojer et al., 2003; Kawauchi et al., 2013).

Both RpdA (class 1) and HdaA (class 2), acting in multiprotein complexes, were shown to contribute to the majority of total HDAC activity in *A. nidulans*, with HdaA being the predominant one (Trojer et al., 2003; Tribus et al., 2005). In contrast, HosA and HosB contribute to a negligible portion of total HDAC activity.

### SIRTUINS

The sirtuins are conserved eukaryotic enzymes requiring nicotinamide adenine dinucleotide (NAD^+^) for their activity. Their role in promoting longevity has generated great interest. In *S. cerevisiae*, SIR2 silences transcription at the silent mating type loci and reduces ribosomal DNA recombination and rDNA extrachromosomal circles production, resulting in an extended life span (Wierman and Smith, 2014). *S. cerevisiae* has four additional SIR2-like sirtuins, HST1-4 (Wierman and Smith, 2014). Little is known about sirtuins in filamentous fungi. Putative SIR2-type and HST-type orthologs were identified by sequence alignment (Brosch et al., 2008). In *A. nidulans*, it is evident that a proportion of the total HDAC activity, albeit less important than that attributed to classical HDACs, is NAD^+^-dependent and thus attributable to sirtuins (Trojer et al., 2003).

## ROLE OF HDACs IN *Aspergillus* GROWTH AND ENVIRONMENTAL ADAPTATION

### HdaA

Genetic deletion of *hdaA*, the major contributor to total HDAC activity, was performed in *A. nidulans*, *A. oryzae* and *A. fumigatus* (Tribus et al., 2005; Lee et al., 2009; Kawauchi et al., 2013). A growth defect was only observed in the *A. fumigatus ΔhdaA* strain, but did not result in decreased virulence in a murine model of IA (Lee et al., 2009). Altered responses to osmotic or oxidative stress were observed in *A. nidulans* and *A. oryzae* (Tribus et al., 2005; Kawauchi and Iwashita, 2014). HdaA was also found to have a role in the production of secondary metabolites, which was associated with the transcriptional regulation of two telomere-proximal secondary metabolic gene clusters (the sterigmatocystin and penicillin clusters; Shwab et al., 2007; Lee et al., 2009).

### RpdA

While RpdA was found to be less important than HdaA for total HDAC activity (Trojer et al., 2003) and could be deleted in yeasts (Robbins et al., 2012), it was found to be essential in *A. nidulans* and *A. oryzae*, as well as in the plant pathogenic filamentous fungus *Magnaporthe oryzae* (Izawa et al., 2009; Tribus et al., 2010; Kawauchi et al., 2013). Genetic repression was achieved in *A. nidulans* by substitution of the native *rpdA* promoter by the alcohol dehydrogenase or xylanase inducible promoters and resulted in an important growth and sporulation defect (Tribus et al., 2010). Truncations in the C-terminal portion of *A. nidulans* RpdA showed that a fungal-specific conserved motif was essential for the catalytic activity of the enzyme and for fungal viability (Tribus et al., 2010). Heterologous expression of the C-terminal motif of *A. fumigatus* RpdA was able to restore RpdA functionality in *A. nidulans*, suggesting a common crucial role of this conserved region among *Aspergillus* spp and possibly other filamentous fungi.

### HosA AND HosB

Despite their minor contribution to total HDAC activity in *Aspergillus* spp, functional analyses suggest that HosA (yeast HOS2) is important for growth and virulence in filamentous fungi. Its deletion in *A. oryzae* had more impact on radial growth and sporulation than the *hdaA* deletion and also resulted in altered stress responses (Kawauchi et al., 2013; Kawauchi and Iwashita, 2014). In plant pathogenic fungi (*Fusarium graminearum*, *M. oryzae*, *Cochliobolus carbonum*), HOS2-related enzymes appear as the most important class 2 HDAC for vegetative growth, sporulation and virulence (Baidyaroy et al., 2001; Izawa et al., 2009; Ding et al., 2010; Li et al., 2011). Indeed, Hos2 (HosA ortholog) was found to contribute to an important part of total HDAC activity in *M. oryzae* (Ding et al., 2010), which was not the case for *A. nidulans* (Trojer et al., 2003).

Genetic deletion of *hosB* (the HOS3 type HDAC) did not result in significant phenotypic consequences in *A. nidulans* and *A. oryzae*, confirming its minor contribution to total HDAC activity although this HDAC seems to have some additive role in metabolite production (Tribus et al., 2005; Shwab et al., 2007; Kawauchi et al., 2013; Kawauchi and Iwashita, 2014).

### OTHER HDACs

Genetic deletion of 6 sirtuins of *A. oryzae* was performed and only HstD/AoHst4 was associated with a significant role in fungal growth, sporulation, stress responses and production of secondary metabolites via the regulation of LaeA (Kawauchi et al., 2013; Kawauchi and Iwashita, 2014).

## THE KEY ROLE OF HDACs IN REGULATING HEAT SHOCK PROTEIN 90

In addition to histones, an increasing number of proteins have been identified as substrates of HDACs, leading to the use of the more general terminology of lysine deacetylases (KDAC; Glozak et al., 2005; Choudhary et al., 2009). Many of these proteins belong to categories that are important for fungal growth and virulence, such as transcription factors, cytoskeletal proteins, and molecular chaperones (Choudhary et al., 2009). Among them, Hsp90 emerges as a key player for which the relationship to HDACs has been recently described. Hsp90 was shown to be essential for fungal survival and to be an important trigger of antifungal resistance to both azole and echinocandin classes and possibly to amphotericin B (Cowen and Lindquist, 2005; Cowen et al., 2009; Singh et al., 2009; Lamoth et al., 2012, 2014b; Blum et al., 2013). In *A. fumigatus*, Hsp90 governs the basal resistance to echinocandins including the paradoxical effect of caspofungin, a compensatory mechanism of the cell wall resulting in decreased caspofungin antifungal activity at increased concentrations (Wiederhold, 2007; Lamoth et al., 2014b). Pan-HDAC inhibitors (depsipeptide, hydroxamic acid analogs) induced acetylation of Hsp90 in human cancer cells, which prevented the binding of Hsp90 to client proteins (Yu et al., 2002; Nimmanapalli et al., 2003). Human HDAC6, a class 2b HDAC related to fungal HDA1, deacetylates Hsp90 and modulates its activity (Bali et al., 2005; Kovacs et al., 2005). Other HDACs of class 2, such as HDAC1 and HDAC10, are also involved in the acetylation of Hsp90 in human (Park et al., 2008; Zhou et al., 2008). Lysine 294 (K294) of human Hsp90 was identified as a key acetylation site for Hsp90 function (Scroggins et al., 2007). Several additional acetylation sites have been recovered in human Hsp90 (Yang et al., 2008; Mollapour and Neckers, 2012).

The role of HDACs in the control of fungal Hsp90 was first demonstrated in *S. cerevisiae* by genetic or pharmacologic inhibition of RPD3 and HDA1, which resulted in the abrogation of Hsp90-dependent azole resistance and impaired interaction of Hsp90 with multiple client proteins (Robbins et al., 2012). Lysine 27 (K27) was found to be acetylated in yeast Hsp90 after deletion of both HDA1 and RPD3 (Robbins et al., 2012). We detected acetylation at K271 (K294 in human) in *A. fumigatus* Hsp90 after treatment with the HDAC inhibitor trichostatin A (TSA; Lamoth et al., 2014c). Mutational analyses actually suggest that both K27 and K271 (K270 in yeast) are important for Hsp90 function. Mutations of both sites resulted in impaired Hsp90 function in *S. cerevisiae* and *A. fumigatus* and were associated with decreased virulence in a murine model of IA (Robbins et al., 2012; Lamoth et al., 2014c). However, while K270 appears to be the predominant site in yeast, K27 seems to be more important in *A. fumigatus*. An acetylation-mimetic mutation (lysine to alanine) of this residue was sufficient to affect Hsp90 function. This effect could be reversed by a deacetylation-mimetic mutation (lysine to arginine) of K27, suggesting that this site must be deacetylated for proper Hsp90 function (Lamoth et al., 2014c). Mutation of K27 alone also resulted in increased susceptibility to both caspofungin and voriconazole in *A. fumigatus* (Lamoth et al., 2014c). However, neither K27 nor K270 mutations were able to decrease azole resistance of an *erg3* mutant of *S. cerevisiae*, while this effect could be achieved by treatment with TSA and by genetic deletion of both RPD3 and HDA1 (Robbins et al., 2012). Thus, the role of HDACs in governing Hsp90 function in antifungal resistance may differ among fungi.

## THE ANTIFUNGAL ACTIVITY OF HDAC INHIBITORS

The fact that TSA, an organic antibiotic produced by *Streptomyces hygroscopicus*, and its hydroxamate analogs display some antifungal activity is well-known (Tsuji et al., 1976). Although these compounds are known to be broad-spectrum inhibitors of both class 1 and 2 HDACs (with the exception of HOS3-like HDAC), their precise mode of action against fungi remains poorly elucidated. HDAC inhibitors were shown to considerably modify the profile of secondary metabolites produced by *Aspergillus* spp., although the impact of this effect on virulence is unclear (Henrikson et al., 2009; Konig et al., 2013). Recent studies suggest that their antifungal effect mainly results from the acetylation and inhibition of Hsp90 (Robbins et al., 2012; Lamoth et al., 2014c). HDACs inhibitors have raised considerable interest because of their potential as anticancer therapy and are undergoing rapid development in the pharmaceutical industry (West and Johnstone, 2014). Some of them also display anti-parasitic activity against *Plasmodium falciparum* and *Toxoplasma gondii* (Strobl et al., 2007; Trenholme et al., 2014).

Trichostatin A has minimal antifungal activity against *C. albicans*, although it potentiates the activity of azoles by a mechanism which is supposed to be essentially mediated via Hsp90 inhibition and associated with loss in the upregulation of *ERG* and *CDR* genes in response to azoles (Smith and Edlind, 2002; Robbins et al., 2012). TSA alone appears to be more active against molds. We have observed a 50 and >90% growth inhibition of the wild-type *A. fumigatus* strain AF293 at TSA concentrations of 1 and 4 μg/ml, respectively (Lamoth et al., 2014c). TSA also had variable antifungal activity against clinical isolates of *Aspergillus* spp. and other pathogenic non-*Aspergillus* molds, and was particularly active against the azole-resistant *A. ustus* and the multi-resistant *Scedosporium prolificans* isolates (Lamoth et al., 2015). TSA also exhibited synergistic activity with caspofungin against some *Aspergillus* spp. and with the Hsp90 inhibitor geldanamycin against *Rhizopus* spp. (Lamoth et al., 2014c, 2015). The differential effect of TSA against *C. albicans* and *A. fumigatus* may be related to different patterns of HDAC activity in these fungi. The better antifungal activity of TSA against *A. fumigatus* is consistent with the finding that genetic compromise of *hdaA* and *rpdA* had a much greater impact on fungal growth and survival in *Aspergillus* spp. compared to deletion of their orthologs in yeast (Lee et al., 2009; Tribus et al., 2010; Robbins et al., 2012).

The effect of TSA against *A. fumigatus* had some similitude with that observed after acetylation-mimetic mutations or genetic repression of *hsp90*, including a growth and conidiation defect and hypersensitivity to geldanamycin (Lamoth et al., 2014c). Moreover, TSA enhanced the antifungal activity of caspofungin and abolished the paradoxical effect, which is a hallmark of Hsp90 inhibition. However, TSA did not potentiate the effect of voriconazole against *A. fumigatus*, which contrasts with previous reports in yeasts showing a positive interaction of TSA with azoles but not with echinocandins (Smith and Edlind, 2002; Robbins et al., 2012). Indeed, differences in Hsp90-dependent pathways of azole and echinocandin resistance between *C. albicans* and *A. fumigatus* have been previously outlined (Lamoth et al., 2014a). Overall, these data support that the antifungal effect of TSA is largely mediated via indirect inhibition of Hsp90. However, considering the role of HDACs in transcriptional regulation, metabolite production, and activation of multiple non-histone proteins, it is probable that other mechanisms are involved.

Trichostatin A was well tolerated in pharmacokinetic murine models, but is rapidly metabolized (half-life of 5–10 min; Sanderson et al., 2004). Numerous novel HDAC inhibitors are currently under investigation for the treatment of cancer in clinical and preclinical trials (West and Johnstone, 2014). Vorinostat (suberoylanilide hydroxamic acid, SAHA) is a TSA analog which has an extended half-life and has been approved by the food and drug administration (FDA) for the treatment of cutaneous T cell lymphoma (Duvic et al., 2007). The antifungal activity of SAHA and other hydroxamate analogs remains to be investigated. Although these compounds have been well tolerated in clinical trials (Duvic et al., 2009; Fouladi et al., 2010), some rare and possibly dose-dependent hematologic adverse events (granulocytopenia, thrombocytopenia) may counterbalance their potential benefit in the setting of invasive fungal diseases.

A selective HDAC inhibitor of fungal HOS2, MGCD290 (MethylGene Inc., Montreal, QC, Canada) was tested against clinical isolates of yeasts and molds (Pfaller et al., 2009). MGCD290 alone displayed some *in vitro* antifungal activity against *Candida* spp. and other yeasts, but was inactive against filamentous fungi. Most importantly, MGCD290 exhibited synergism with azoles (fluconazole, voriconazole and posaconazole) against both yeast and mold species. The combination of MGCD290 and fluconazole was recently tested for the treatment of vulvovaginal candidiasis in a randomized phase II study but did not demonstrate a benefit compared to fluconazole monotherapy (Augenbraun et al., 2013).

## CONCLUSION AND PERSPECTIVES

In filamentous fungi, HDACs are involved in multiple processes contributing to virulence via transcription control and functional regulation of important proteins (Figure 1). HDAC inhibition thus represents an interesting alternative antifungal approach to current strategies targeting the cell wall or membrane. The antifungal effect of the HDAC inhibitor TSA and its analogs against *A. fumigatus* seems to be mainly achieved via the acetylation and inhibition of Hsp90 (Lamoth et al., 2014c). This molecular chaperone is essential for virulence and triggers stress responses and resistance to the most important antifungal classes (Lamoth et al., 2014a). HDAC inhibitors have demonstrated a potential as antifungal monotherapy *in vitro*, as well as adjunctive therapies to enhance the effect of existing drugs against *A. fumigatus* (Pfaller et al., 2009; Lamoth et al., 2014c, 2015). Their potential as anti-cancer therapy is generating an intense research activity in drug development with several compounds currently investigated in clinical and pre-clinical trials (West and Johnstone, 2014). There is a great opportunity to harness these recent advances, and gain in-depth understanding of fungal HDACs, to pursue novel and more effective antifungal strategies against IA.

### Conflict of Interest Statement

The authors declare that the research was conducted in the absence of any commercial or financial relationships that could be construed as a potential conflict of interest.
